# Molecular Basis of Adipose–Cardiac Crosstalk in Cardiovascular Diseases: From Mechanisms to Therapeutic Opportunities

**DOI:** 10.3390/biom16081093

**Published:** 2026-07-27

**Authors:** Siqi Gan, Qixuan Zhang, Chan Zhang, Li Yan, Yue Yin, Heng Ma, Zihui Zhang

**Affiliations:** 1 Xi’an Key Laboratory of Stem Cell and Regenerative Medicine, School of Life Science and Technology, Northwestern Polytechnical University, Xi’an 710072, China; 2 Department of Physiology and Pathophysiology, School of Basic Medicine, Fourth Military Medical University, Xi’an 710032, China

**Keywords:** cardiovascular adipose tissue, adipose–cardiac crosstalk, adipokines, cardiovascular disease, extracellular vesicles, inflammation, metabolic remodeling

## Abstract

Cardiovascular diseases remain the leading cause of mortality worldwide and are closely associated with obesity and metabolic dysfunction. Adipose tissue is now recognized as a heterogeneous endocrine and immunometabolic organ that actively communicates with the cardiovascular system through adipokines, inflammatory mediators, metabolites, and extracellular vesicles. Under physiological conditions, adipose–cardiac crosstalk contributes to metabolic and cardiovascular homeostasis, whereas adipose tissue dysfunction promotes inflammation, fibrosis, endothelial injury, and cardiac remodeling. This review summarizes the heterogeneity of adipose depots and their secretomes, discusses the molecular mechanisms underlying adipose–cardiac communication, and highlights their contributions to atherosclerosis, heart failure, hypertension, diabetic cardiomyopathy, and atrial fibrillation. We further discuss emerging biomarkers, therapeutic strategies, and precision medicine approaches targeting the adipose–cardiac axis. Understanding depot-specific signaling networks may facilitate the development of novel diagnostic and therapeutic interventions for cardiometabolic diseases.

## 1. Introduction

In spite of significant progress in managing risk factors and the creation of targeted treatments, cardiovascular diseases (CVDs) continue to be the primary cause of death globally [1]. The growing prevalence of obesity and metabolic dysfunction has further accelerated this burden, placing cardiometabolic disease at the center of a global public health challenge. Elevated body mass index (BMI) is a major modifiable risk factor for cardiovascular disease, with consistent evidence from large prospective cohorts and Mendelian randomization studies linking higher BMI to increased cardiovascular morbidity and mortality [2,3,4]. However, the so-called “obesity paradox” observed in heart failure (HF) and coronary heart disease highlights the limitations of BMI as a sole indicator of cardiovascular risk [5,6]. Emerging evidence suggests that the quality of adipose tissue is more critical than its quantity. This emphasizes that factors such as depot distribution, biological function, and secretory activity are more pertinent to cardiovascular outcomes than total fat mass alone [7,8].

This shift in perspective has redefined adipose tissue as a heterogeneous endocrine-immunometabolic organ with significant implications for cardiovascular function. Visceral, subcutaneous, epicardial, and perivascular adipose depots exhibit considerable variability in their developmental origins, cellular composition, and paracrine/endocrine characteristics, thereby exerting distinct effects on cardiovascular homeostasis [9,10]. Through the release of adipokines, inflammatory mediators, lipids, and extracellular vesicles (EVs), adipose tissue maintains ongoing bidirectional crosstalk with the heart. Under physiological conditions, this adipose–cardiac axis supports metabolic equilibrium and preserves cardiac integrity; however, in the setting of obesity and metabolic stress [11], adipose tissue becomes dysfunctional, adopting a pro-inflammatory and pro-fibrotic phenotype that drives myocardial remodeling, endothelial damage, and electrical instability [12,13,14,15]. Conversely, the stressed heart can reshape adipose tissue biology through natriuretic peptides and other cardiomyokines, creating a maladaptive feedback loop. This interorgan crosstalk has emerged as a unifying mechanism underlying atherosclerosis, HF, hypertension, diabetic cardiomyopathy (DCM), and atrial fibrillation (AF) [16]. However, the systemic regulatory framework, disease-specific effects of different adipose depots, and translational potential remain inadequately elucidated. In this review, we summarize the heterogeneity of adipose tissue and the dynamics of its secretome, explore the molecular foundations of adipose–cardiac communication, and evaluate its pathological and therapeutic significance across various CVDs, with a particular emphasis on translational opportunities for precision cardiometabolic medicine.

This review is intended for basic and translational researchers, clinicians, cardiologists, endocrinologists, and other healthcare professionals interested in adipose–cardiac communication, cardiometabolic medicine, and CVD mechanisms. By integrating mechanistic insights with clinical evidence, this review aims to support readers in understanding the pathophysiological relevance, translational potential, and therapeutic implications of adipose–cardiac crosstalk in cardiovascular and metabolic diseases.

## 2. Methods

### 2.1. Literature Search Strategy

This review was designed as a structured narrative review rather than a systematic review or meta-analysis. A structured literature search was conducted to identify studies relevant to adipose–cardiac crosstalk and its roles in CVDs. PubMed/MEDLINE, Web of Science, and Scopus were searched from database inception to 1 June 2026. The search strategy combined terms related to adipose tissue, interorgan communication, molecular mediators, cardiometabolic dysfunction, and CVDs. The principal search terms included “adipose tissue”, “adipose–cardiac crosstalk”, “adipose–heart axis”, “cardiometabolic disease”, “obesity”, “metabolic dysfunction”, “insulin resistance”, “epicardial adipose tissue”, “perivascular adipose tissue”, “visceral adipose tissue”, “brown adipose tissue”, “adipokines”, “inflammatory mediators”, “extracellular vesicles”, “gut microbiota”, “inflammation”, “fibrosis”, “endothelial dysfunction”, “cardiac remodeling”, “metabolic remodeling”, “coronary artery disease”, “atherosclerosis”, “heart failure”, “hypertension”, “diabetic cardiomyopathy”, and “atrial fibrillation”. These terms were combined using the Boolean operators “AND” and “OR” as appropriate. Additional studies were identified by manually screening the reference lists of eligible articles and key reviews.

### 2.2. Eligibility Criteria and Evidence Synthesis

Original mechanistic studies, preclinical investigations, clinical studies, systematic reviews, and meta-analyses were considered eligible if they addressed adipose tissue biology, adipose-derived mediators, adipose–cardiac communication, CVD mechanisms, biomarkers, or therapeutic implications. Studies were excluded if they were unrelated to adipose–cardiac crosstalk, focused exclusively on obesity without cardiovascular relevance, lacked sufficient mechanistic or clinical information, duplicated previously reported findings, or were available only as conference abstracts, editorials, letters, protocols, or non-peer-reviewed reports.

Given the broad mechanistic and translational scope of the review and the heterogeneity of the available evidence, a narrative synthesis was performed. Priority was given to original mechanistic studies, high-quality clinical investigations, systematic reviews, and meta-analyses. Evidence supported by direct cardiac or vascular functional findings was distinguished from broader associations with adipose dysfunction and from emerging hypotheses requiring further validation. Atherosclerosis, heart failure, hypertension, diabetic cardiomyopathy, and atrial fibrillation were selected as representative disease contexts because they encompass vascular, myocardial, metabolic, hemodynamic, and electrophysiological manifestations of adipose–cardiac axis dysfunction. Studies were organized according to adipose depot, molecular mediator, signaling pathway, disease phenotype, biomarker potential, and therapeutic relevance. No formal quantitative assessment of study quality or risk of bias was performed.

## 3. Heterogeneity of Adipose Tissue and Its Secretome

Adipose tissue is now recognized as a dynamic metabolic and endocrine organ. Owing to profound heterogeneity in anatomical distribution, blood perfusion, immune microenvironment, and secretory phenotype across distinct adipose depots, these tissues exert divergent effects on cardiovascular homeostasis and pathogenesis [17].

### 3.1. Classification and Functional Disparities of Adipose Subtypes

Adipose tissue comprises three main subtypes: white adipose tissue (WAT), brown adipose tissue (BAT), and beige/brite adipose tissue, each exhibiting distinct physiological functions and responses to pathological stress. WAT functions as the primary site for energy storage and acts as a key endocrine organ that modulates lipid and glucose metabolism, adipokine secretion, and the balance of immune-inflammatory responses. In the context of obesity, WAT experiences maladaptive remodeling, which is characterized by adipocyte hypertrophy, chronic inflammation, and fibrosis; this process further exacerbates lipotoxicity and insulin resistance, both of which are core characteristics of type 2 diabetes mellitus [18].

BAT specializes in adaptive thermogenesis facilitated by uncoupling protein 1. Its mass and activity diminish with age and the accumulation of excess body fat [19]. In addition to its thermogenic function, BAT secretes bioactive molecules termed batokines, which regulate cardiac function and confer cardioprotective effects. A growing body of evidence links reduced BAT activity to the onset and progression of CVDs, particularly pathological myocardial injury [20,21].

In addition to systemic adipose depots, epicardial adipose tissue (EAT) and perivascular adipose tissue (PVAT) play crucial roles in regulating local factors that maintain cardiac and vascular homeostasis [8,22]. EAT is in direct physical contact with the myocardium and shares the coronary microcirculation, thereby enabling it to exert significant paracrine and vasocrine effects [23,24]. Under conditions of obesity or metabolic stress, EAT exhibits a pro-inflammatory secretory profile that promotes local inflammation, myocardial damage, and the progression of CVDs [25]. Conversely, PVAT generally exerts beneficial vascular effects due to widespread production of different vasodilator mediators. However, as metabolic disease progresses, PVAT adopts a more pro-oxidant and pro-inflammatory phenotype, driving the production of reactive oxygen species (ROS), vascular remodeling, and atherosclerotic lesion formation [26]. The observations presented herein suggest that EAT and PVAT may serve as valuable therapeutic targets in the management of cardiometabolic diseases. Notably, adipocytes do not exhibit a unidirectional pathway for differentiation.

Beige/brite adipocytes exhibit significant phenotypic plasticity, enabling their interconversion between white adipocyte-like and brown adipocyte-like states in response to various metabolic and environmental signals. This dynamic conversion complicates the classification of adipose tissue and allows for alterations in adipose subtypes, thereby modifying their function. Collectively, these adipose depots contribute to adipose–cardiac communication through the secretion of diverse bioactive mediators, whose molecular actions and cardiovascular effects are summarized in Table 1.

### 3.2. Adipokine Profiles and Secretomic Characteristics

**Figure 1:**
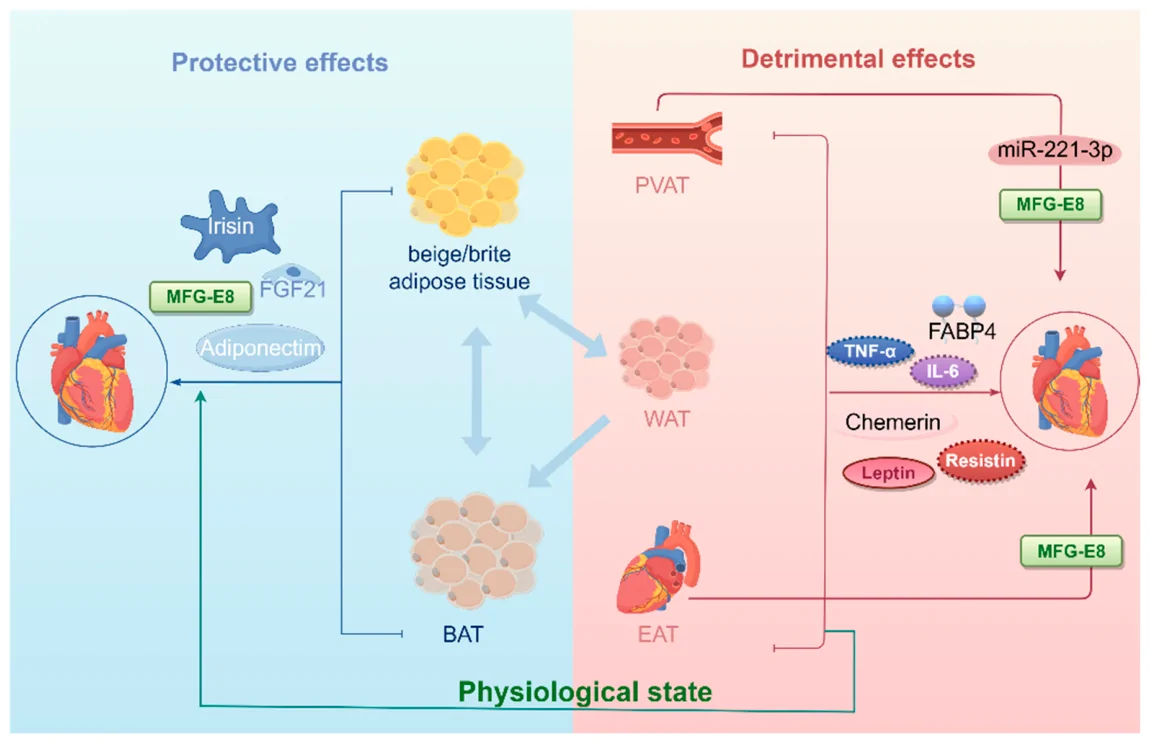
Heterogeneity of adipose tissue and its secretome in cardio-metabolic regulation. WAT, BAT, and beige/brite adipose tissue exhibit distinct metabolic and endocrine functions. WAT primarily serves as an energy reservoir and secretes adipokines that regulate metabolism and inflammation. In contrast, BAT is integral to adaptive thermogenesis and secretes cardioprotective batokines. Beige/brite adipose tissue demonstrates phenotypic plasticity, capable of interconverting between WAT- and BAT-like states. Cardiovascular-specific depots, such as EAT and PVAT, exert local modulatory effects on the heart and vascular system. Protective factors, including adiponectin, irisin, FGF21, and MFG-E8, enhance cardiac function, whereas dysregulated depots in the context of obesity or metabolic stress secrete pro-inflammatory cytokines (e.g., TNF-α, IL-6), pathogenic adipokines (e.g., leptin, resistin), and EVs (e.g., miR-221-3p), contributing to myocardial injury, vascular dysfunction, and atherosclerosis. Created by the authors using Figdraw 2.0.

The endocrine and paracrine functions of adipose tissue are primarily mediated by its secretome, which serves as the molecular foundation for adipose–cardiac signaling. This secretome includes classical adipokines, mediators of inflammation-metabolism crosstalk, novel signaling molecules, and EVs (Figure 1).

#### 3.2.1. Classical Adipokines

Leptin is a prominent pro-inflammatory adipokine that stimulates the production of tumor necrosis factor-α (TNF-α), interleukin-6 (IL-6), and interleukin-1β (IL-1β). Additionally, it promotes vasoconstriction and the migration and proliferation of vascular smooth muscle cells (VSMC). Through the leptin receptor-dependent activation of cyclin D1 and the mitogen-activated protein kinase (MAPK) pathway, leptin contributes to VSMC hyperproliferation and the formation of atherosclerotic plaques [30].

In contrast, adiponectin exerts anti-inflammatory and vasculoprotective effects in a receptor-dependent manner. AdipoR1 primarily activates AMP-activated protein kinase (AMPK), enhancing energy metabolism and insulin sensitivity. Meanwhile, AdipoR2 preferentially stimulates the peroxisome proliferator-activated receptor-α (PPARα) pathway, leading to a reduction in circulating lipids [27]. A decrease in adiponectin levels is a hallmark of metabolic and cardiovascular disorders.

Resistin is implicated in insulin resistance, cardiac fibrosis, and type 2 diabetes. It activates downstream inflammatory pathways, including Janus kinase 2 (JAK2)/signal transducer and activator of transcription 3 (STAT3) and JNK/c-Jun signaling by binding to cysteine-rich protein 1 and toll-like receptor 4. This activation exacerbates insulin resistance and pathological fibrosis [34,35].

Irisin, initially identified as an exercise-induced myokine, is also secreted by adipose tissue and acts as a myokine–adipokine mediator. Through integrin αV/β5, irisin promotes the browning of WAT, enhances glucose and lipid metabolism, and mitigates cardiac injury and insulin resistance [36,37,38].

Visfatin is another pro-inflammatory adipokine implicated in metabolic dysregulation and atherosclerosis. It promotes the proliferation of VSMC through nicotinamide mononucleotide-dependent extracellular signal-regulated kinase (ERK) 1/2 and p38 signaling pathways, thereby accelerating vascular remodeling and plaque development [50].

Beyond the well-characterized classical adipokines, numerous novel adipose-derived signaling factors have been identified. Their exploration is pivotal for comprehensively decoding the complex network of adipose–cardiac crosstalk and uncovering additional actionable therapeutic targets for cardiometabolic disorders.

#### 3.2.2. Inflammation-Metabolism Crosstalk Factors

Under conditions of chronic nutrient excess and adipose tissue expansion, the adipose secretome transitions from metabolic regulation to inflammatory signaling, thereby linking adipose dysfunction to systemic metabolic imbalance [51]. In this context, IL-6 and TNF-α impair insulin receptor signaling, induce systemic insulin resistance, and directly damage cardiomyocytes and vascular endothelial cells, thereby accelerating HF and atherosclerosis [11,52,53]. Similarly, chemerin promotes atherosclerotic progression through the activation of the MAPK/ERK pathway and the induction of pro-inflammatory cytokines such as IL-6 and TNF-α [54]. Together, these mediators establish a state of low-grade chronic inflammation, known as metaflammation, which is central to metabolic CVDs.

#### 3.2.3. Emerging Adipose-Derived Signaling Molecules

Among the newly recognized adipose-derived mediators, fatty acid-binding protein 4 (FABP4) has emerged as a critical pathogenic link between systemic lipotoxicity and cardiovascular injury. Elevated FABP4 secretion exacerbates macrophage-driven inflammation and aggravates cardiovascular structural and functional impairment, underscoring its potential as both a biomarker and a therapeutic target [42,55]. In contrast, milk fat globule-EGF factor 8 (MFG-E8) functions as a protective component of the adipose secretome, facilitating efferocytosis during atherogenesis and thereby mitigating maladaptive inflammation and tissue injury [56]. These findings highlight the dual functional roles of the adipose secretome, which encompasses both pathogenic and protective signals. Fibroblast growth factor 21 (FGF21) is a significant endocrine regulator associated with the adipose–cardiac axis. While primarily synthesized in the liver, FGF21 is also expressed in adipose tissue and the heart, particularly under conditions of metabolic stress. As a stress-responsive metabolic hormone, FGF21 mitigates inflammation, oxidative stress, and fibrosis [45]. Mechanistically, it operates through fibroblast growth factor receptors and the co-receptor β-Klotho, stimulating adiponectin secretion and enhancing insulin sensitivity [46,47]. Consequently, FGF21 contributes to cardiometabolic homeostasis and indirectly influences adipose–cardiac interactions.

#### 3.2.4. EVs

In addition to soluble adipokines, EVs are key mediators of adipose–cardiovascular communication [57]. Adipose tissue contributes substantially to the circulating EV pool, and this contribution appears to increase under obese conditions [58,59]. Although EVs are often classified by size as small EVs, typically <200 nm, or medium/large EVs, typically >200 nm, these categories do not necessarily indicate distinct biogenetic origins [60]. Functionally, adipose-derived EVs can transfer proteins, lipids, and regulatory RNAs, including miRNAs and lncRNAs, to vascular and cardiac recipient cells. EVs from inflammatory or metabolically dysfunctional adipose tissue promote vascular inflammation by increasing endothelial VCAM-1 expression and leukocyte adhesion [14,61,62]. Consistent with the context-dependent effects summarized in Figure 1, BAT-derived EV miRNAs, including miR-125b-5p, miR-128-3p, and miR-30d-5p, may exert protective effects by attenuating oxidative stress [63], whereas miR-27b-3p from VAT-derived EVs and miR-221-3p from PVAT-derived EVs drive endothelial inflammation, atherosclerosis, and VSMC proliferation [48,49]. Overall, the cardiovascular effects of adipose-derived EVs depend on the depot of origin, cellular source, pathological context, molecular cargo, and recipient cell type, but causal and subtype-specific evidence linking adipose-derived EVs to CVDs remains limited.

### 3.3. Clinical Relevance of Adipose Depot Diversification

The heterogeneity of adipose tissue extends beyond a mere biological characteristic; it emerges as a clinically significant determinant of cardiometabolic risk. In cases of obesity and CVDs, the disrupted communication between adipose tissue and the heart functions not only as a pathogenic mechanism but also as a therapeutic target. Notably, the secretory signatures unique to each adipose depot may provide more valuable biomarkers for risk stratification and precision medicine compared to traditional indicators such as BMI or total fat mass. Therefore, integrating the specific characteristics of different adipose depots into clinical assessments could enhance diagnostic precision, improve patient stratification, and facilitate more targeted prevention and treatment of metabolic-related CVDs.

## 4. Molecular Mechanisms of Adipose–Cardiac Crosstalk

**Figure 2:**
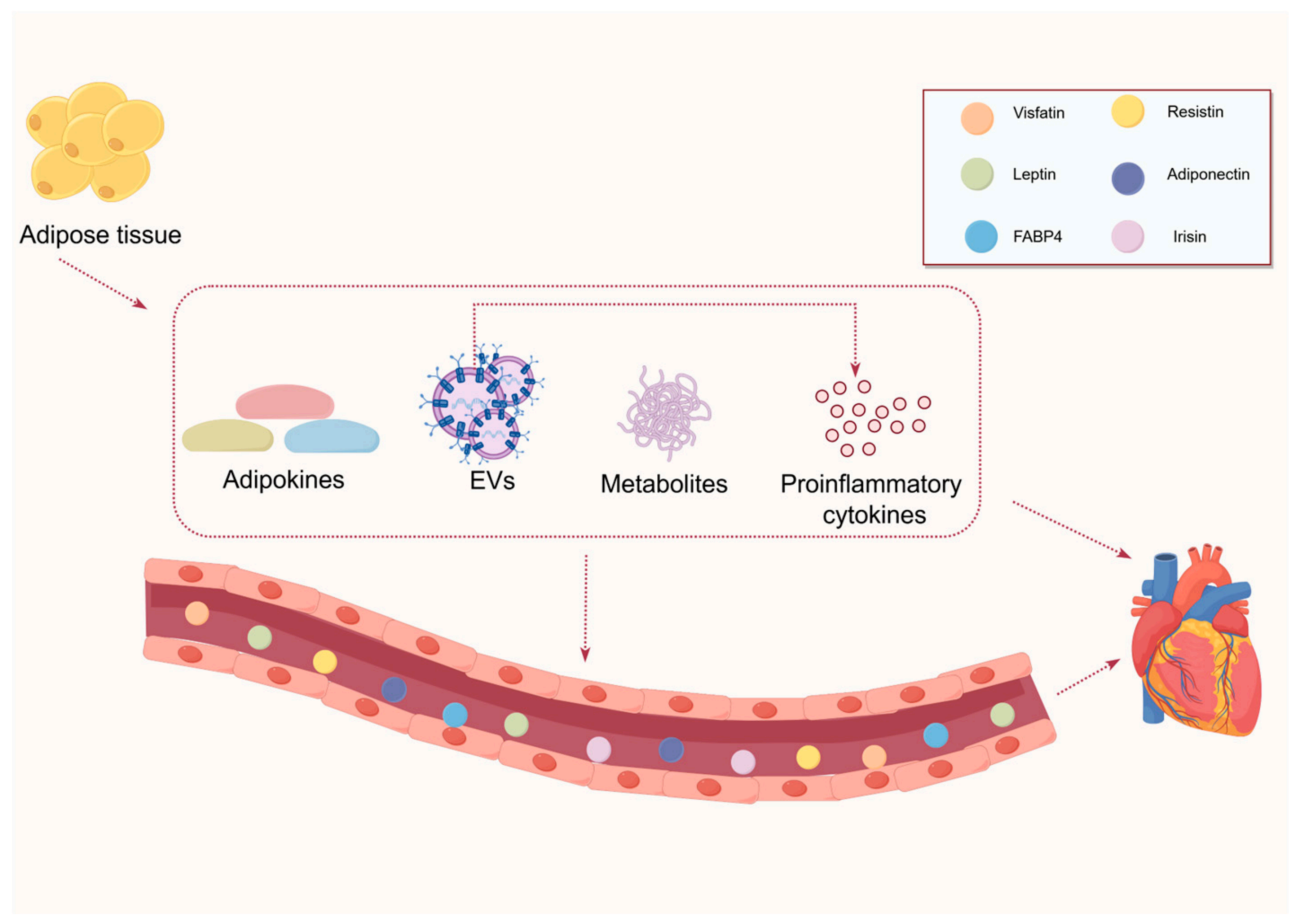
Key molecular mechanisms underlying adipose–cardiac crosstalk. Adipose tissue secretes adipokines, EVs, metabolites, and proinflammatory cytokines, which travel through the bloodstream to the heart. These secretions, including visfatin, leptin, resistin, adiponectin, FABP4, and irisin, play a role in regulating inflammation, metabolism, and cardiovascular health, potentially impacting the heart. EVs may also act as pro-inflammatory mediators. Created by the authors using Figdraw.

Adipose–cardiac crosstalk constitutes a multi-layered network that regulates systemic cardiometabolic homeostasis. Various inter-organ communication processes arise from classical adipokine signaling and inflammatory responses, extending to metabolite-induced injury and newly identified non-canonical pathways. Clarifying these mechanisms is crucial for understanding the pathogenesis of adipose–cardiac crosstalk and for developing targeted therapies. Therefore, this section delineates the key molecular events underpinning adipose–cardiac crosstalk from four distinct mechanistic perspectives. Figure 2 provides a schematic overview of the key molecular mechanisms underlying adipose–cardiac crosstalk.

### 4.1. Adipokine-Mediated Signaling Pathways

Adiponectin is a cardioprotective adipokine that activates AMPK and PPARα via its receptors AdipoR1 and AdipoR2, thereby promoting fatty acid oxidation and mitochondrial homeostasis in cardiomyocytes. Additionally, adiponectin enhances vascular endothelial function by stimulating endothelial nitric oxide synthase (eNOS) activity, which subsequently increases nitric oxide (NO) production. In conditions of obesity and insulin resistance, reduced levels of adiponectin disrupt myocardial metabolism and elevate oxidative stress, leading to endothelial dysfunction, fibrosis, and the progression of obesity-related or diabetic cardiomyopathies [28,29].

Leptin activates the JAK2/STAT3 pathway via its receptor Ob-R, resulting in the upregulation of hypertrophy-associated genes and the induction of cardiac structural remodeling [31]. In the vasculature, leptin inhibits the expression of peroxisome proliferator-activated receptor γ (PPARγ) through the ERK1/2 and nuclear factor kappa-B (NF-κB) signaling pathways, thereby promoting the proliferation and migration of VSMCs. This process accelerates vascular remodeling and atherosclerosis, collectively increasing the risk of HF and obesity-related CVDs [32,33].

FABP4, derived from adipocytes and macrophages, plays a significant role in cardiac remodeling by enhancing lipid transport and inflammatory signaling. Under conditions of lipid or pressure overload, FABP4 promotes the ubiquitination and degradation of PPARγ; this suppression triggers cellular activation and the deposition of extracellular matrix components in cardiac fibroblasts, ultimately leading to myocardial fibrosis and dysfunction [43,44].

Irisin binds to integrin αV/β5, activating the AMPK/FAK/Akt signaling pathway. This activation suppresses NF-κB-mediated inflammatory responses, decreases the expression of pro-inflammatory cytokines such as IL-6 and TNF-α, and mitigates endothelial cell injury and vascular VSMC proliferation [39,40,41]. Consequently, these effects alleviate vascular inflammation and hinder the progression of atherosclerosis.

Visfatin activates the NF-κB signaling pathway, leading to an increased expression of MMP-2/9, ICAM-1, and VCAM-1. This activation promotes leukocyte adhesion and vascular inflammatory responses, thereby contributing to endothelial dysfunction and significantly influencing the progression of atherosclerosis [64,65].

In conclusion, the proteins produced by both protective and harmful adipose tissue play a crucial role in the communication between adipose tissue and the heart. Disruption of metabolic equilibrium alters the nature of the adipokine secretome, shifting it toward pro-inflammatory and profibrotic states, which contribute to the development and progression of CVDs.

### 4.2. Adipose-Related Inflammatory Cascades

In addition to their roles in hormone production, dysfunctional adipocytes and immune cells present within adipose tissue also serve as crucial inflammatory mediators. As metabolic stress occurs, the adipose secretome experiences notable changes, initiating a “molecular storm” that conveys inflammatory signals from peripheral depots to the heart.

Chemerin activates the MAPK/ERK signaling pathway via its receptor CMKLR1, resulting in an increased production of pro-inflammatory cytokines, such as IL-6 and TNF-α. These cytokines promote endothelial inflammation, enhance monocyte adhesion, and stimulate the proliferation and migration of VSMCs, thereby contributing to vascular inflammation and remodeling [66].

Inflammatory mediators derived from adipose tissue play a pivotal role in bridging systemic metabolic inflammation and cardiac injury. Among these mediators, chemokines secreted by adipose tissue, particularly monocyte chemoattractant protein-1 (MCP-1), facilitate the recruitment of CCR2^+^ monocytes to cardiac tissue through the activation of the CCR2 signaling pathway. Within this localized inflammatory microenvironment, these monocytes differentiate into pro-inflammatory macrophages. The infiltrating macrophages further release pro-inflammatory cytokines such as TNF-α, IL-1β, and IL-6, thereby amplifying local inflammatory responses in the myocardium. This process contributes to cardiomyocyte dysfunction, extracellular matrix remodeling, and myocardial fibrosis [67]. Consequently, the resultant chronic inflammatory microenvironment in cardiac tissue is regarded as a significant mechanism driving the development and progression of obesity-related CVDs.

Enhanced pro-inflammatory signaling is a hallmark pathological feature of the dysregulated adipose–cardiac axis, wherein the Nod-Like Receptor Family, Pyrin Domain Containing 3 (NLRP3) inflammasome serves as a central molecular switch. The activation of the NLRP3 inflammasome promotes the maturation and secretion of IL-1β and interleukin-18, which directly impair cardiomyocyte contractile function and induce cardiomyocyte apoptosis, ultimately exacerbating ischemic and inflammatory myocardial injury [68]. This inflammatory cascade clearly illustrates how aberrant adipose tissue inflammation remotely triggers myocardial damage, thereby establishing a robust mechanistic link between obesity and the progression of CVDs.

Suppressor of cytokine signaling 3 (SOCS3) is a crucial inflammatory feedback regulator induced by IL-6 and IL-1β. It mediates insulin resistance in both adipose tissue and the heart by directly inhibiting insulin receptor substrate signaling and downstream insulin-mediated metabolic pathways [69,70,71]. This finding highlights the dual regulatory role of adipose tissue in connecting metabolic disorders and cardiac inflammatory injury, creating a vicious cycle that exacerbates the progression of cardiometabolic diseases.

In summary, the inflammatory cascades associated with adipose tissue represent a significant “remote-trigger” mechanism that translates systemic metabolic stress into localized damage within the heart.

### 4.3. Metabolite-Mediated Mechanisms

In addition to adipokines and inflammatory mediators, the release of bioactive metabolites from dysfunctional adipose tissue is believed to have a detrimental impact on cardiac function. This phenomenon represents a significant non-adipokine mechanism of adipose–cardiac crosstalk. Under the metabolic stress associated with obesity, adipose tissue alters substrate handling and catabolic capacity, thereby contributing circulating metabolites that exert chronic stress on the myocardium.

Circulating levels of specific metabolic substances, particularly branched-chain amino acids (BCAAs), have increasingly been linked to significant complications. These amino acids, which include leucine, isoleucine, and valine, are associated with insulin resistance, systemic metabolic derangements, and myocardial lipotoxicity as their levels rise. Research indicates that dysfunctional adipose tissue, which results in reduced catabolism of BCAAs, is a major contributor to their accumulation in the body. A decrease in the activity of branched-chain α-ketoacid dehydrogenase is one of the primary factors underlying this effect [72]. Elevated circulating levels of BCAAs increase metabolic stress in cardiomyocytes due to impaired clearance. Studies have shown that excessive BCAAs can activate the mTOR signaling pathway, disrupt insulin signaling, and ultimately reduce myocardial metabolic flexibility, thereby contributing to the onset and progression of HF [72,73].

In addition to amino acid metabolites, lipid intermediates produced by adipose tissues represent a significant pathogenic link between obesity and CVDs. In states of obesity or chronic low-grade inflammation, increased adipose lipolysis elevates circulating levels of free fatty acids (FFAs) and lipotoxic metabolites. These lipotoxic species accumulate in cardiac muscle cells, disrupting cellular metabolism. For instance, palmitoyl-CoA interferes with the regulation of intracellular calcium and mitochondrial oxidative phosphorylation, ultimately impairing myocardial contractility and inducing cardiomyocyte death [74]. Moreover, ceramides and diacylglycerols activate stress-responsive signaling pathways, such as protein kinase C, exacerbating cardiac insulin resistance and contributing to myocardial metabolic dysfunction and structural remodeling [75,76].

In conclusion, our data identify adipose-derived metabolites as critical effectors in the crosstalk between adipose tissue and the heart. These metabolites link adipose dysfunction to myocardial injury through sustained metabolic and signaling perturbations. Although the transport of BCAAs and lipid metabolites to the heart, as well as their context-dependent pathological actions, are emerging as actionable therapeutic targets, they remain incompletely understood. Furthermore, the heterogeneity of patient metabolic profiles and dietary exposures affects the successful clinical translation of metabolite-targeted therapies, highlighting the need for phenotype-guided and personalized metabolic interventions.

### 4.4. Non-Canonical and Emerging Regulatory Mechanisms

Adipose tissue plays a significant role in cardiac remodeling through novel non-canonical pathways, including EVs communication, microbial metabolites, and epigenetic modifications. These pathways provide additional layers of inter-organ communication, effectively linking adipose dysfunction to myocardial hypertrophy, fibrosis, and metabolic remodeling.

Building on the secretomic role of adipose-derived EVs discussed above, EV-mediated transfer of inflammatory proteins and regulatory RNAs represents an emerging mechanism linking adipose dysfunction to cardiac remodeling. Under obese conditions, EV cargoes such as Sonic hedgehog and inflammation-related miRNAs, including miR-34 and miR-155, may promote macrophage activation, M1-like polarization, and cytokine release [77,78,79], thereby contributing to inflammatory myocardial remodeling. In addition, EV-derived non-coding RNAs may influence cardiomyocyte stress responses, extracellular matrix turnover, and fibroblast activation. For instance, EV-associated lncRNA LINC00636 may attenuate myocardial fibrosis through the miR-450a-2-3p/MAPK1 pathway [80], suggesting that individual EV-associated RNA cargos may have distinct roles in cardiac fibrotic remodeling.

The gut microbiota also plays a significant role in shaping adipose–cardiac communication through the gut–adipose–heart axis. Trimethylamine N-oxide (TMAO) exacerbates myocardial fibrosis and inflammation by activating TGF-β signaling and the NLRP3 inflammasome [81,82], whereas short-chain fatty acids (SCFAs) generally confer cardioprotective effects by suppressing inflammation through G protein-coupled receptor activation and histone deacetylase inhibition [83].

Epigenetic alterations in adipose tissue, particularly DNA methylation, significantly impact adipokine secretion and inflammatory signaling. Abnormal methylation patterns of adiponectin, leptin, and pro-inflammatory genes can intensify adipose inflammation and fibrosis-related signaling, thereby contributing to adverse cardiac remodeling [84,85].

Taken together, these non-canonical pathways broaden the mechanistic understanding of adipose–heart crosstalk and may present novel therapeutic avenues. However, the interactions among these pathways and their specific roles at different disease stages require further investigation. To provide a structured overview, Table 2 summarizes the key mechanistic domains, molecular events, representative mediators, cardiac consequences, and potential therapeutic targets discussed in this section.

## 5. Pathophysiological Roles of the Adipose–Cardiac Axis in CVDs

**Figure 3:**
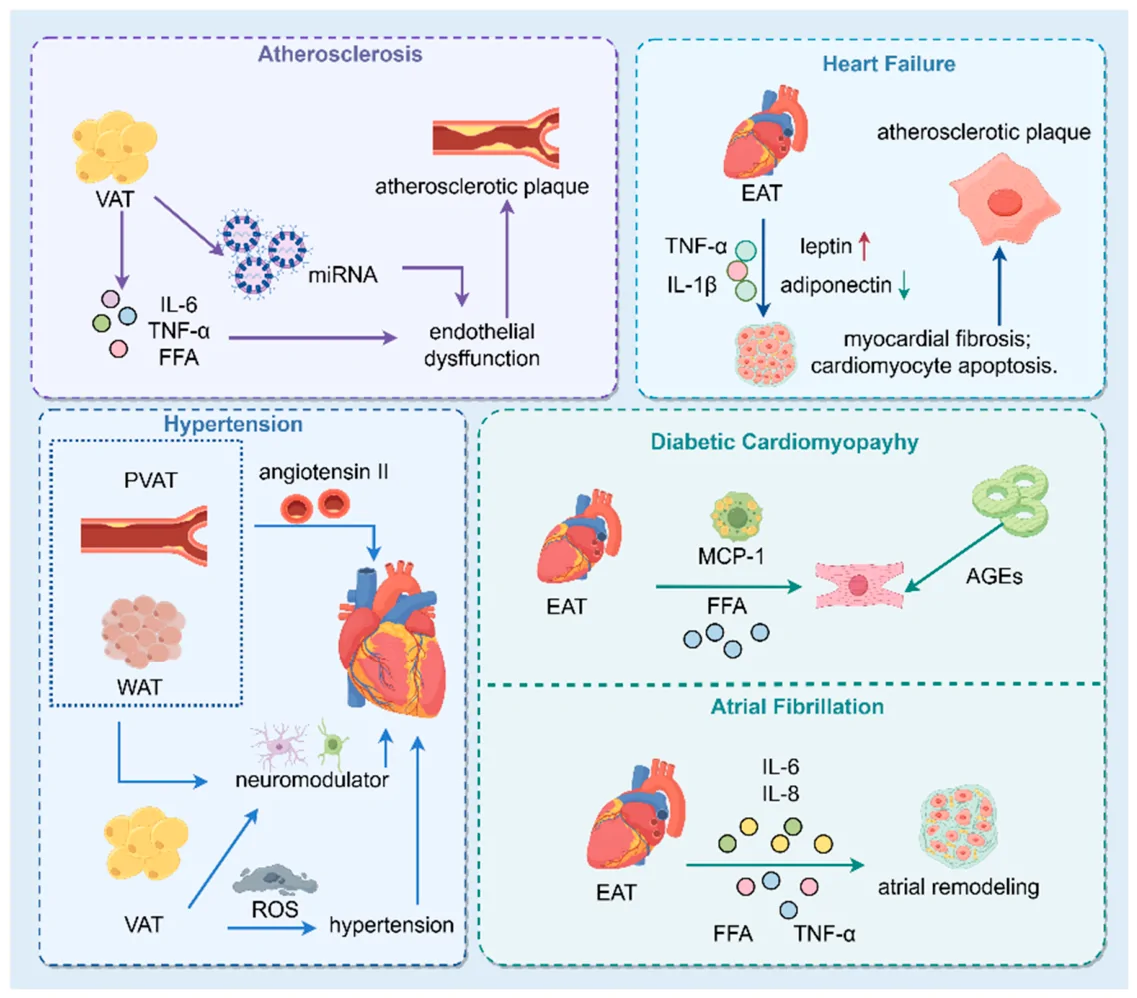
Roles of adipose–cardiac crosstalk in major CVDs. Schematic overview of the pathological roles of adipose–cardiac crosstalk in CVDs. In atherosclerosis, adipose-derived cytokines, FFAs, and miRNAs promote endothelial dysfunction, lipid deposition, and plaque progression. In HF, EAT shifts toward a pro-inflammatory and pro-fibrotic phenotype, driving myocardial remodeling, apoptosis, and metabolic dysfunction. In hypertension-associated hypertrophy, VAT contributes to renin–angiotensin system activation, oxidative stress, and sympathetic overactivity, thereby increasing afterload and ventricular remodeling. In DCM, lipotoxicity, chronic inflammation, and AGE–RAGE signaling promote fibrosis and diastolic dysfunction. In AF, EAT-mediated inflammation, fatty infiltration, and electrical remodeling increase arrhythmogenic susceptibility and recurrence. Created by the authors using Figdraw.

Adipose–cardiac crosstalk exerts heterogeneous effects across various CVDs. Depot-specific adipose dysfunction, altered secretory profiles, and local paracrine signaling contribute to distinct pathological processes in atherosclerosis, HF, hypertension, DCM, and AF. As a crucial upstream regulator of cardiometabolic disease, adipose tissue significantly influences the initiation, progression, and prognosis of CVDs through inflammatory, metabolic, neurohumoral, and epigenetic mechanisms (Figure 3).

### 5.1. Atherosclerosis

In obesity, VAT promotes atherosclerosis through inflammatory activation and atherogenic lipid remodeling. TNF-α activates NF-κB signaling in endothelial cells, which upregulates the expression of ICAM-1 and VCAM-1 [86]. IL-6 further impairs eNOS activity via the IL-6R/gp130-JAK/STAT3 pathway, thereby reducing NO bioavailability and enhancing oxidative stress, which aggravates endothelial dysfunction [87]. Collectively, these adipose-derived inflammatory signals accelerate early plaque formation [87,88,89]. Meanwhile, excess free FFAs released from VAT increase hepatic triglyceride synthesis and very-low-density lipoprotein secretion, contributing to dyslipidemia and plaque progression [90,91,92]. Additionally, adipose-derived EVs carrying miR-27b-3p further exacerbate endothelial injury and vascular inflammation, promoting atherosclerosis progression [48].

### 5.2. HF

Under pathological conditions, EAT transitions from a cardioprotective to a pro-inflammatory and pro-fibrotic phenotype. This shift is associated with the activation of the NF-κB, JAK/STAT, and MAPK signaling pathways, which lead to an increase in IL-1β, TNF-α, and other mediators that promote cardiomyocyte apoptosis, myocardial fibrosis, reduced ventricular compliance, and the progression of HF [22,52,91,93]. An imbalance in adipokines further exacerbates cardiac injury, characterized by elevated levels of leptin and decreased levels of adiponectin [94,95]. In high-fat diet-induced obese rats, leptin derived from pericardial adipose tissue induces cardiomyocyte apoptosis through JAK2/STAT3 activation, mitochondrial injury, calcium overload, and Na+/K+-ATPase inhibition [95]. Conversely, adiponectin alleviates myocardial ischemia–reperfusion injury through AMPK activation [96]. Moreover, excessive fatty acid supply from dysfunctional EAT intensifies myocardial lipid accumulation and metabolic stress, leading to impaired energy production and contractile dysfunction [97,98,99].

### 5.3. Hypertension

In obesity-related hypertension, adipose tissue contributes to elevated blood pressure and cardiac hypertrophy through activation of the renin–angiotensin–aldosterone system (RAAS), oxidative stress, and sympathetic overactivity [100]. Adipose-derived angiotensin II promotes vasoconstriction and increases peripheral resistance. In fructose-fed rat models, the local activation of the RAS in VAT is increased. Elevated levels of angiotensin II lead to increased peripheral vascular resistance and elevated blood pressure [101]. Following a sustained increase in systemic pressure, the left ventricle undergoes concentric remodeling and dilation. This condition, referred to as hypertrophy, may precipitate the onset of HF.

Additionally, oxidative stress contributes to the pathogenesis of these conditions. In cases of obesity and metabolic disease, dysfunction of PVAT increases the production of ROS, which promotes the degradation of NO. This degradation inhibits guanylate cyclase activation, reduces cyclic guanosine monophosphate (cGMP) levels, and impairs vascular relaxation [102]. In high-fat diet-induced obese mice, reduced NO bioavailability causes endothelial dysfunction in PVAT and accelerates systemic hypertension [103].

In addition, hypertrophic adipose tissue may enhance sympathetic nerve activity through factors such as nerve growth factor, which activates TrkA-dependent signaling pathways including MAPK, PI3K/AKT, and PLC-γ, thereby promoting sympathetic nerve growth and plasticity [104]. Enhanced sympathetic drive activates renal sympathetic output and the RAAS, further increasing vascular resistance and blood pressure [104]. In high-fat diet-induced obese rats, this sympathetic activation is closely associated with myocardial hypertrophy, and β-adrenergic blockade attenuates both hypertrophy and myocardial inflammation [105].

### 5.4. DCM

DCM is primarily driven by lipotoxicity, chronic inflammation, and fibrosis mediated by advanced glycation end products (AGEs). Under conditions of chronic hyperglycemia and insulin resistance, the expansion of WAT and EAT leads to an elevation in circulating FFAs. Excess FFAs enter cardiomyocytes via CD36, enhancing β-oxidation; however, when mitochondrial capacity is exceeded, lipid intermediates accumulate, resulting in mitochondrial overload, ROS generation, and subsequent lipotoxic damage [106]. Concurrently, excessive FFA utilization activates protein kinase C-related pathways, disrupts insulin signaling, inhibits GLUT4-mediated glucose uptake, and further suppresses myocardial glucose utilization [107]. Consistent with this, ^1^H-MRS studies have shown that myocardial lipid accumulation in patients with type 2 diabetes is positively correlated with impaired diastolic function [108].

Inflammatory signaling exacerbates myocardial remodeling. EAT-derived MCP-1 recruits circulating monocytes, which differentiate into pro-inflammatory M1 macrophages within the myocardium, sustaining a chronic inflammatory microenvironment that promotes cardiomyocyte death and extracellular matrix remodeling [109]. Simultaneously, hyperglycemia-driven AGEs bind to RAGE, activating NF-κB, MAPK, and TGF-β/Smad signaling pathways, which enhance ROS production and pro-fibrotic responses, ultimately accelerating myocardial stiffening and diastolic dysfunction [110,111]. Lipotoxicity, inflammation, and AGEs collectively contribute to metabolic disturbances that may progress to HF with preserved ejection fraction (HFpEF) [112]. Notably, the DAPA-LVH trial showed that dapagliflozin significantly reduced left ventricular mass in patients with type 2 diabetes and left ventricular hypertrophy, accompanied by improvements in insulin resistance, systemic inflammation, and adiposity [113]. These findings support the potential of SGLT2 inhibitors to attenuate adverse cardiac remodeling in DCM.

### 5.5. AF

AF is a prevalent cardiac arrhythmia, with its initiation and maintenance closely linked to the structural and electrophysiological remodeling of the atria. Moreover, obesity is recognized as an independent risk factor for AF [114]. EAT promotes AF by secreting inflammatory mediators and directly infiltrating adjacent atrial myocardium. Cytokines such as IL-6, IL-8, and TNF-α activate the TGF-β1/Smad signaling pathway, stimulate atrial fibroblast proliferation, and promote fibrosis, thereby increasing atrial structural heterogeneity and facilitating electrical remodeling [115,116].

Imaging studies have shown that EAT volume is significantly associated with the incidence and recurrence of AF [117]. In prospective non-randomized studies, preoperative left atrial EAT volume was closely related to post-ablation AF recurrence, supporting its clinical predictive value [117]. Mechanistically, FFAs and inflammatory mediators released from EAT inhibit L-type calcium channels, reduce calcium current density, and increase abnormal automaticity, thereby promoting early afterdepolarizations and triggered activity [118,119]. In addition, adipocyte infiltration disrupts cardiomyocyte coupling and creates regions of heterogeneous conduction, which, in conjunction with fibrosis, provide a substrate for reentry and AF maintenance [116].

Consequently, EAT-driven atrial remodeling is a central mechanism in the pathogenesis of AF. However, the molecular link between EAT inflammation and atrial electrical remodeling requires further elucidation, and standardized methods for EAT quantification are still needed before its broader clinical application.

## 6. Clinical and Translational Implications

**Figure 4:**
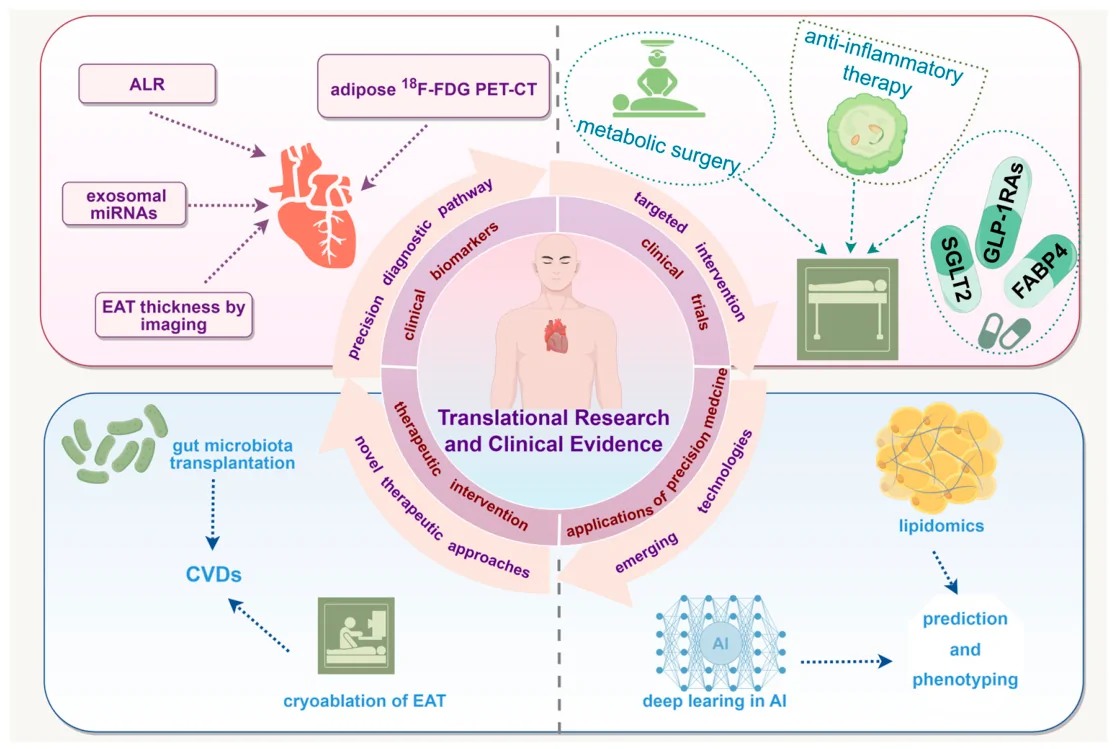
Clinical and translational advances targeting the adipose–cardiac axis; Overview of clinical biomarkers, imaging tools, and therapeutic strategies related to adipose–heart crosstalk. Circulating markers such as the adiponectin/leptin ratio and EVs miRNAs (e.g., miR-146a, miR-126) reflect adipose dysfunction and cardiovascular risk. Multimodal imaging, including ^18^F-FDG PET-CT, cardiac CT, and MRI, enables quantification of adipose inflammation and EAT volume, providing incremental prognostic value. Pharmacological interventions (e.g., GLP-1RAs, SGLT2 inhibitors, anti–IL-1β therapy), metabolic surgery (e.g., RYGB), and emerging device-based or microbiota-targeted approaches modulate adipose inflammation and remodeling. Integration of lipidomics and AI-based imaging analysis further supports precision risk stratification and individualized cardiovascular therapy. Created by the authors using Figdraw.

The dysregulated adipose–cardiac axis has emerged as a clinically relevant target in cardiometabolic disease. Current research hotspots include circulating and imaging biomarkers, targeted pharmacological and surgical interventions, precision medicine approaches, and device-based therapies, as outlined in Figure 4.

### 6.1. Biomarkers for Risk Stratification

The adiponectin/leptin ratio (ALR) is the sole circulating marker that reflects adipose endocrine dysfunction. A large community-based prospective cohort study revealed that a lower ALR is associated with a higher incidence of HF. Furthermore, mediation analysis indicated that ALR may partially mediate the relationship between obesity-related indices, such as BMI, waist-to-hip ratio, and visceral fat area, and HF [120].

The imaging modality can evaluate the metabolic activity of adipose tissue and low-grade inflammation using ^18^F-FDG PET-CT [121,122]. In cases of familial combined hyperlipidemia, an increase in visceral fat and a decrease in adiponectin levels were correlated with heightened ^18^F-FDG uptake in VAT [121]. A single-arm prospective study demonstrated that the metabolic volume of BAT increased following 12 weeks of treatment with extended-release exenatide, indicating that PET-CT can effectively monitor therapies targeting BAT [122].

EAT is another significant biomarker. Non-invasive analysis of adipose distribution and heterogeneity can be achieved through cardiac magnetic resonance imaging (MRI) [123], while cardiac computed tomography (CT) remains the primary technique for quantifying EAT volume and radiodensity, which reflect inflammatory and fibrotic remodeling [124,125,126]. In a cohort study, EAT volume measured by CT was found to independently predict MI beyond coronary calcium scores and traditional risk factors in the Heinz Nixdorf Recall cohort [127].

The diagnostic potential of exosomal miRNAs is significant. Serum exosomal miR-146a is associated with systemic inflammation and effectively distinguishes acute coronary syndrome from healthy controls, as demonstrated by ROC analysis [128]. Furthermore, exosomal miR-126 shows a positive correlation with the severity of coronary stenosis in cases of acute myocardial infarction [129].

Despite these advances, the absence of standardized ALR cutoffs, the cost and radiation burden associated with PET-CT and CT, as well as the need for standardized exosomal miRNA assays, continue to restrict their clinical implementation.

### 6.2. Targeted Therapeutic Strategies

GLP-1 receptor agonists (GLP-1RAs) and SGLT2 inhibitors have emerged as pivotal therapies for type 2 diabetes, with cardiovascular benefits likely attributable in part to reduced adipose inflammation and improved adipokine profiles [130,131,132,133,134,135,136]. Across multiple trials, SGLT2 inhibitors, including empagliflozin, canagliflozin, and dapagliflozin, consistently reduced HF hospitalization [130,131,132]. GLP-1RAs such as semaglutide, liraglutide, and dulaglutide, improved glycemic control and reduced major adverse cardiovascular events, with effects partly independent of weight loss [133,134,135,136]. FABP4 has also emerged as a metabolic target; anagliptin has been shown to reduce circulating FABP4 levels in statin-treated patients with type 2 diabetes [137].

The CANTOS trial provided direct evidence that inflammation is a modifiable cardiovascular target, as IL-1β inhibition with canakinumab reduced recurrent cardiovascular events after myocardial infarction [138]. Given that adipose tissue is a major source of IL-1β and related cytokines, these findings support the notion of targeting adipose inflammation therapeutically.

Metabolic surgery further underscores the pathogenic role of adipose dysfunction. Roux-en-Y gastric bypass (RYGB) reduces visceral adiposity, improves insulin resistance, and lowers inflammatory markers, thereby improving cardiac remodeling [139,140]. The Swedish Obese Subjects study demonstrated that metabolic surgery significantly reduced cardiovascular mortality [141]. Additionally, randomized trials have shown reductions in BMI, HbA1c, HOMA-IR, TC, TG, LDL-C, TNF-α, and hs-CRP following RYGB [142].

The clinical benefits of these approaches are evident; however, their broader application is constrained by an incomplete understanding of the underlying mechanisms, the costs and infection risks associated with biologics, and the invasiveness of surgical interventions. Representative studies supporting the clinical relevance of adipose–cardiac axis dysfunction are summarized in Table 3.

### 6.3. Precision Medicine Approaches

Multi-omics analyses are enhancing the risk stratification of adipose–cardiac axis dysfunction. Nuclear magnetic resonance-based plasma lipidomics refines the phenotyping of coronary artery disease [145]. In a pediatric cohort, glycerophosphocholine metabolites such as PC14:1/0:0 were strongly associated with visceral fat, insulin resistance, and dyslipidemia [146]. Another investigation identified 18 lipid signatures that exhibited a strong correlation with the incidence of HF, particularly with cholesteryl esters [147]. These findings suggest that lipidomic signatures may complement traditional cardiovascular risk markers.

The precise assessment of cardiometabolic risk is significantly enhanced through the use of artificial intelligence (AI) [148]. AI models that integrate clinical variables, imaging, and multi-omics data have the potential to improve predictions of cardiovascular risk and treatment responses [149,150]. An analysis conducted across multiple centers demonstrated that automated imaging relies on deep learning models. Furthermore, the measurement of EAT using CT independently predicts all-cause mortality after adjusting for CVDs. Both the volume of EAT and its radiodensity are associated with mortality and the incidence of myocardial infarction [151]. However, current lipidomic and AI-based studies are limited by sample size, population heterogeneity, interpretability, and cross-center generalizability.

### 6.4. Device-Based Interventions and Microbiota-Targeted Therapies

#### 6.4.1. Device-Based Interventions

Device-based therapies are particularly relevant in AF, where EAT is strongly associated with the disease burden [152,153]. Advances in catheter ablation techniques, especially intracardiac echocardiography-guided cryoablation, have significantly improved procedural precision and patient tolerability [153,154]. In a prospective study involving 52 AF patients undergoing cryoballoon pulmonary vein isolation, all participants achieved successful isolation; 97% exhibited complete electrical disconnection, 71% maintained sinus rhythm or showed clinical improvement, and 56% remained free from AF recurrence without the use of antiarrhythmic drugs [155].

#### 6.4.2. Gut Microbiota Modulation

Concurrently, the gut–adipose–cardiac axis has emerged as a potential therapeutic target. Gut microbiota influences systemic metabolism, immunity, and adipose inflammation, thereby impacting atherosclerosis and acute coronary syndrome [156]. In animal studies, fecal microbiota transplantation led to a reduction in IL-6 and TNF-α levels, consequently attenuating adipose inflammation [157]. Conversely, the transfer of pathogenic microbiota accelerated atherosclerosis in germ-free mice. These findings support the modulation of the microbiota as a potential non-pharmacological strategy [156,157,158].

In summary, the clinical translation of the adipose–cardiac axis is advancing from biomarker discovery to targeted intervention and precision medicine. Future studies should focus on the validation of standardized biomarkers, the development of therapies informed by mechanistic insights, and the utilization of multicenter datasets to support individualized treatment approaches.

## 7. Challenges and Future Perspectives

**Figure 5:**
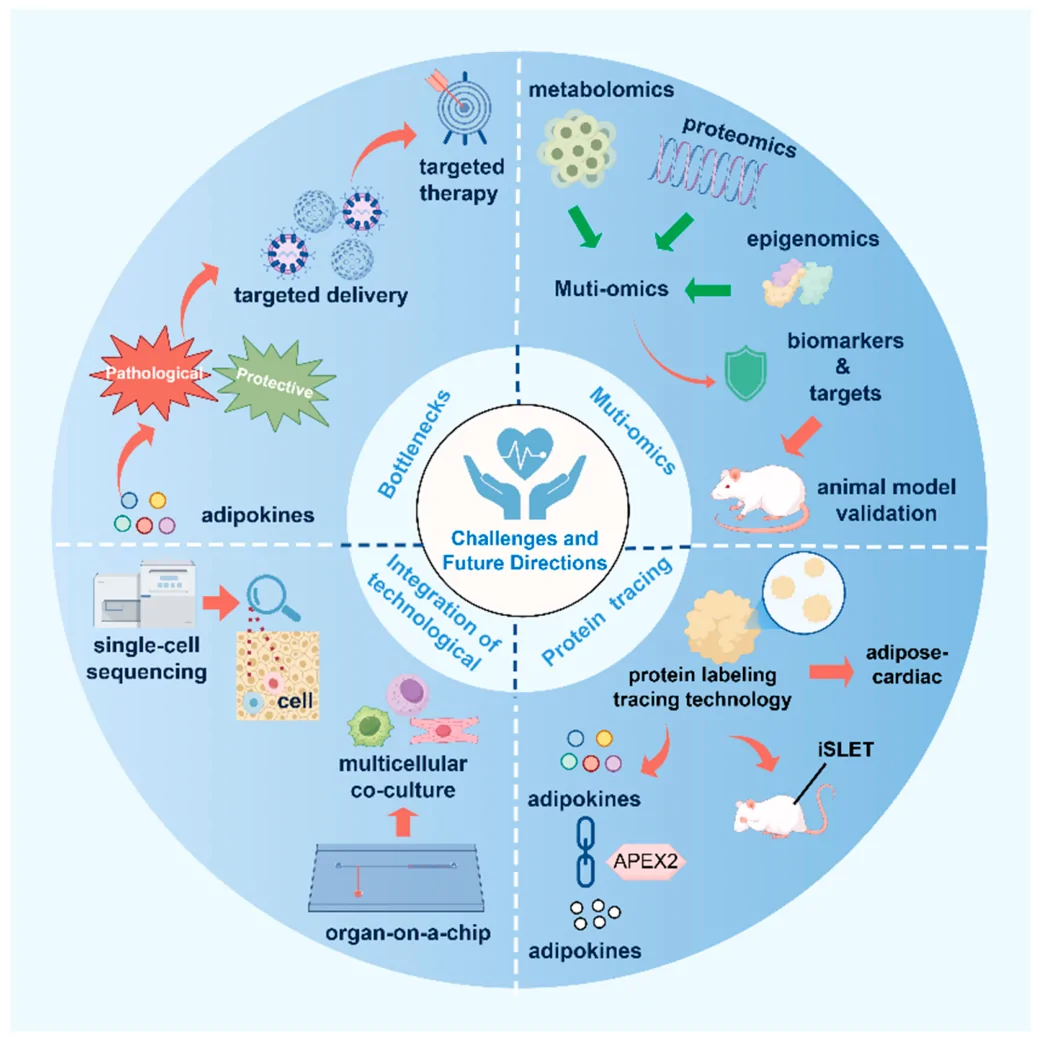
Challenges and Future Directions in Adipose–Cardiac Crosstalk Research. The diagram illustrates key components in understanding the interactions between adipose tissue and the heart. It highlights the roles of adipokines in both pathological and protective contexts. The chart also emphasizes the integration of advanced technologies like single-cell sequencing, multicellular co-culture, organ-on-a-chip models, and protein labeling tracing to address bottlenecks and enhance therapeutic targeting. Future directions focus on multi-omics approaches, animal model validation, and the use of innovative protein tracing techniques, such as iSLET, to explore adipose–cardiac communication and potential biomarkers for disease treatment. Created by the authors using Figdraw.

Despite significant advancements in elucidating the molecular and clinical relevance of the adipose–cardiac axis, substantial gaps remain between mechanistic discoveries and their clinical applications. These challenges primarily arise from the biological plasticity of adipose tissue, the context-dependent effects of adipokines, and the limitations inherent in current experimental and analytical methodologies. To overcome the above issues, it is imperative to implement phenotype-guided therapies, high-resolution technology, an integrated multi-omics framework, and protein labeling tracing technology (Figure 5).

### 7.1. Bottlenecks in Translational Medicine

A central challenge in translation is the context-dependent action of adipokines, which complicates the implementation of straightforward agonist- or antagonist-based strategies. Leptin exemplifies this complexity: in cases of obesity, chronic hyperleptinemia fosters vascular inflammation, smooth muscle cell proliferation, insulin resistance, and adverse cardiac remodeling. Conversely, during acute myocardial ischemia, leptin may exert cardioprotective effects by mitigating lipotoxicity and reducing cardiomyocyte apoptosis [159,160]. A similar paradox is observed with adiponectin. While it is predominantly anti-inflammatory and insulin-sensitizing, elevated adiponectin levels in chronic kidney disease are unexpectedly linked to increased cardiovascular risk and poor prognosis [96,161]. These observations suggest that the actions of adipokines are significantly influenced by metabolic status, comorbidities, disease stage, and the local tissue environment.

This complexity limits conventional systemic adipokine-targeted therapy due to poor tissue specificity and potential off-target effects. Targeted delivery platforms, including polymeric nanoparticles, engineered EVs, and lipid nanoparticles, may help overcome this limitation by facilitating site-specific modulation in cardiomyocytes or EAT while minimizing systemic exposure [162,163,164]. However, improved delivery alone is insufficient. Future studies should identify context-specific regulatory nodes, incorporate metabolic phenotyping and disease staging into study design, and validate phenotype-tailored interventions in stratified clinical trials.

### 7.2. Integration of Advanced Technological Approaches

Due to the highly heterogeneous nature of adipose–cardiac crosstalk in both cellular composition and spatial organization, bulk analyses often fail to capture its complexity. Single-cell RNA sequencing can identify disease-related cell subsets and rare populations within adipose and cardiac tissues [165]. When integrated with spatial transcriptomics, this technique further delineates the localization of these cells within the tissue architecture and reveals localized intercellular signaling networks [166]. In the context of atherosclerosis, this combined strategy elucidates the spatial distribution and phenotypic states of endothelial cells, smooth muscle cells, and macrophages within plaques, thereby providing a more precise framework for therapeutic targeting [167].

However, transcriptomic approaches alone cannot replicate the functional dynamics at the tissue level. Organ-on-a-chip technology offers a complementary platform by reconstructing multicellular microenvironments and enabling real-time monitoring of electrophysiology, metabolic flux, and mechanical activity [168]. A human WAT-on-a-chip model has already been established to study adipocyte responses to drugs and inflammatory stimuli [169]. Future systems that integrate human adipose and cardiac microtissues within microfluidic platforms may facilitate direct investigations of adipose–cardiac communication under controlled hormonal, inflammatory, and hemodynamic conditions [170].

Despite their potential, these technologies continue to encounter significant limitations, including high costs, low throughput, challenges in multi-dimensional data integration, and inadequate simulation of systemic immune and neurohumoral regulation. Enhancing scalability and physiological relevance is crucial for their broader translational application.

### 7.3. Multi-Omics-Driven Systemic Research Paradigms

Metabolomics, proteomics, and epigenomics have identified multiple candidate mediators of adipose–cardiac dysfunction, including metabolites such as TMAO and BCAAs, inflammatory proteins like IL-6, non-coding RNAs such as miRNAs, and epigenetic modifications that encompass DNA methylation and histone alterations [171,172]. However, single-omics analyses often yield only fragmented insights into this inter-organ network.

Integrative multi-omics approaches are more adept at capturing the complexity of adipose–cardiac crosstalk by linking molecular alterations across various biological layers. For instance, a clinical study that combined proteomic and metabolomic network analyses identified thyroxine as a central metabolite in HF progression, suggesting its role in adipose–cardiac metabolic regulation [173]. Such approaches transcend descriptive profiling, facilitating the identification of key regulatory hubs and mechanistic pathways.

Nevertheless, current multi-omics studies are still constrained by small cohort sizes, population heterogeneity, and inadequate cross-validation among transcriptomic, proteomic, metabolomic, and epigenomic datasets. Furthermore, bioinformatic tools for integrating high-dimensional data remain underdeveloped. Future research should aim to establish large, well-phenotyped longitudinal cohorts, develop robust analytical pipelines for multi-omics integration, and combine discovery-based analyses with tissue-specific gene editing or functional validation platforms. Such initiatives may facilitate a translational framework that links biomarker discovery, target identification, and mechanistic validation, thereby enhancing precision prevention and treatment of cardiometabolic diseases.

### 7.4. Protein Labeling Tracing Technology

Protein labeling tracing technology is a powerful tool for investigating interorgan crosstalk, providing direct, dynamic, and visual insights into molecular cues and signal transduction between different organs. This technology has enabled comprehensive analyses of the mechanisms linking adipose tissue to the heart in adipose–cardiac crosstalk research. Protein labeling tracing allows for the dynamic tracking of the synthesis, secretion, and targeting of functional proteins, including adipokines and cardiomyokines, which contribute to adipose–cardiac crosstalk and cannot be detected through static analyses.

A genetically encoded proximity labeling probe with surface-displayed APEX2 enables high-fidelity in vitro biotinylation and mass spectrometry-based characterization of EV surface proteins, corona proteins, and their interactors [174]. This provides a robust technical tool to advance EV biology research, biomarker discovery, and the development of EV-based therapeutics. The proximity-labeling-based proteomic approach, characterized by a narrow labeling range that targets EV membrane components near specific surface antigens, enables the effective identification of cell-type-specific EVs. This technique can accurately characterize EV-associated proteins and distinguish disease-related EV subsets, highlighting its utility for EV-based research and clinical applications. The in vivo proximity labeling tool iSLET (in situ Secretory protein Labeling via ER-anchored TurboID) addresses the limitations of in vitro and ex vivo models by selectively labeling proteins within the classical secretory pathway [175]. This capacity enables accurate tracking and characterization of tissue-specific secretory proteins in vivo and has been validated in mouse liver, demonstrating efficient labeling and tracking of liver secretory proteins in circulating plasma [176]. Such capabilities are crucial for advancing research on biomarkers and therapeutics related to secretory proteins. Furthermore, the protein labeling techniques for cells and intercellular interactions provide a foundational basis for investigating the target cells affected by which interacting proteins, as well as the mechanisms through which these proteins exert their effects (Figure 5).

## 8. Conclusions

The adipose–cardiac axis has emerged as a crucial framework for understanding the pathogenesis of CVDs, moving beyond the traditional view that regards the heart as an isolated organ. Evidence from epidemiological studies, mechanistic research, and translational investigations indicates that adipose tissue serves not only as an energy reservoir but also as a dynamic regulator of cardiovascular homeostasis and disease progression. Adipose tissue engages in bidirectional communication with the heart through the secretion of adipokines, inflammatory mediators, metabolic substrates, EVs, and the activation of associated signaling pathways. Disruption of this crosstalk promotes the onset and progression of atherosclerosis, HF, AF, and obesity-related cardiomyopathy. Collectively, these findings establish the adipose–cardiac axis as a pivotal mechanistic link in cardiometabolic disease, highlighting its significant potential for the identification of novel diagnostic markers and therapeutic targets.

Despite significant advancements in this field, several critical challenges remain. The biological effects of signals derived from adipose tissue are highly contingent upon the specific physiological or pathological context, and current experimental models inadequately capture the intricate spatial, temporal, and phenotypic characteristics of adipose–cardiac interactions. Further efforts are necessary to refine the characterization of underlying mechanisms, standardize the detection and application of biomarkers, and enhance the validation of translational research. Future studies should prioritize mechanism-based patient stratification and integrate multi-omics technologies, imaging techniques, and functional research approaches. Ultimately, a more comprehensive understanding of the adipose–cardiac axis is likely to transform existing notions of cardiovascular pathophysiology and unlock new possibilities for the precise prevention and treatment of cardiometabolic diseases.

## Figures and Tables

**Table 1:** Major adipose-derived mediators and their molecular actions in adipose–cardiac crosstalk.

Mediator	Source Depot	Molecular Target/Pathway	Cardiovascular Effects	Major CVDs	Refs.
Adiponectin	WAT	AdipoR1/AMPK; AdipoR2/PPARα	Anti-inflammatory; endothelial protection	HF, DCM	[27,28,29]
Leptin	WAT, EAT	Ob-R/JAK2/STAT3; MAPK	Hypertrophy; vascular inflammation	HF, Atherosclerosis	[30,31,32,33]
Resistin	WAT	JAK2/STAT3; JNK	Insulin resistance; fibrosis	DCM	[34,35]
Irisin	BAT, WAT	Integrin αVβ5/AMPK/Akt	Cardioprotection	Atherosclerosis	[36,37,38,39,40,41]
FABP4	WAT, macrophages	PPARγ suppression	Fibrosis; remodeling	HF	[42,43,44]
FGF21	BAT/WAT/ Liver	FGFR1c/β-Klotho	Anti-inflammatory	Multiple CVDs	[45,46,47]
miR-27b-3p	VAT EVs	PPARα inhibition	Endothelial dysfunction	Atherosclerosis	[48]
miR-221-3p	PVAT EVs	VSMC proliferation	Vascular remodeling	Hypertension	[49]

Abbreviations: WAT, white adipose tissue; BAT, brown adipose tissue; EAT, epicardial adipose tissue; VAT, visceral adipose tissue; PVAT, perivascular adipose tissue; HF, heart failure; DCM, diabetic cardiomyopathy; EVs, extracellular vesicles.

**Table 2:** Mechanisms of adipose tissue–cardiac crosstalk and potential therapeutic targets.

Mechanistic Category	Key Molecular Events	Representative Mediators	Major Cardiac Outcomes	Potential Therapeutic Targets	Refs.
Adipokine signaling	Dysregulated adipokine secretion perturbs myocardial metabolic homeostasis, inflammatory responses, and vascular function	Adiponectin, leptin, FABP4, irisin, visfatin	Cardiac hypertrophy, fibrosis, endothelial dysfunction, vascular inflammation	AMPK activators, leptin signaling antagonists, FABP4 inhibitors	[28,29,31,32,33,39,40,41,42,43,44,64,65]
Inflammatory signaling	Chronic adipose inflammation promotes cytokine release, immune-cell recruitment, and inflammasome activation	MCP-1, TNF-α, IL-6, IL-1β, chemerin, NLRP3, SOCS3	Immune-cell infiltration, fibrosis, myocardial injury, insulin resistance	CCR2 inhibitors, anti-IL-1β therapy, NLRP3 inhibitors	[66,67,68,69,70,71]
Metabolic remodeling	Impaired substrate utilization drives myocardial lipotoxicity and metabolic inflexibility	BCAAs, FFAs, palmitoyl-CoA, ceramides, DAG	Insulin resistance, mitochondrial dysfunction, heart failure	Metabolic reprogramming, BCAA catabolism enhancement	[72,73,74,75,76]
Emerging interorgan regulatory mechanisms	EV-mediated communication, microbiota-derived metabolites, and epigenetic remodeling regulate cardiac remodeling and dysfunction	EV-associated miRNAs, lncRNAs, TMAO, SCFAs	Fibrosis, vascular remodeling, metabolic adaptation	EV-based therapy, microbiota modulation, epigenetic intervention	[80,81,82,83,84,85]

Abbreviations: AMPK, AMP-activated protein kinase; BCAAs, branched-chain amino acids; CCR2, C-C chemokine receptor type 2; DAG, diacylglycerol; EVs, extracellular vesicles; FABP4, fatty acid-binding protein 4; FFAs, free fatty acids; IL, interleukin; lncRNAs, long non-coding RNAs; MCP-1, monocyte chemoattractant protein-1; miRNAs, microRNAs; NLRP3, NOD-like receptor family pyrin domain-containing 3; SCFAs, short-chain fatty acids; SOCS3, suppressor of cytokine signaling 3; TMAO, trimethylamine N-oxide; TNF-α, tumor necrosis factor-alpha.

**Table 3:** Clinical and translational evidence of adipose–cardiac axis dysfunction.

Category	Representative Study	Adipose-Related Parameter/Intervention	Major Clinical Implications	Refs.
Circulating adipokine biomarkers	Prospective HF cohort	ALR	Lower ALR was associated with increased heart failure incidence, supporting its utility as a biomarker of adipose endocrine dysfunction.	[120]
Epicardial adipose imaging	Heinz Nixdorf Recall study	EAT volume measured by CT	Higher EAT volume independently predicted myocardial infarction beyond traditional cardiovascular risk factors.	[127]
Visceral adipose inflammation imaging	PET-CT study	VAT FDG uptake	Increased inflammatory activity in VAT was associated with metabolic dysfunction, supporting the clinical relevance of adipose inflammation.	[121]
Inflammatory pathway targeting	CANTOS trial	IL-1β inhibition	Validated inflammation as a therapeutic target for reducing recurrent cardiovascular events.	[138]
Metabolic (bariatric) surgery	SOS study and RYGB trials	Bariatric surgery with visceral fat reduction and inflammatory remodeling	Reduced cardiovascular mortality and an improved cardiometabolic profile, supporting the reversal of adipose dysfunction as a therapeutic strategy.	[141,142]
Emerging metabolic therapies	Representative clinical trials of SGLT2 inhibitors and GLP-1 receptor agonists	Indirect modulation of adipose metabolism	Improved cardiovascular outcomes with potential benefits associated with adipose tissue remodeling and reduced inflammation.	[130,131,132,133,134,135,136,143,144]

Abbreviations: ALR, adiponectin/leptin ratio; CANTOS, Canakinumab Anti-inflammatory Thrombosis Outcomes Study; CT, computed tomography; EAT, epicardial adipose tissue; FDG, 18F-fluorodeoxyglucose; GLP-1RA, glucagon-like peptide-1 receptor agonist; HF, heart failure; IL-1β, interleukin-1 beta; PET-CT, positron emission tomography–computed tomography; RYGB, Roux-en-Y gastric bypass; SGLT2, sodium-glucose cotransporter 2; SOS, Swedish Obese Subjects; VAT, visceral adipose tissue.

## Data Availability

No datasets were generated or analysed during the current study.

## Abbreviations

The following abbreviations are used in this manuscript:

AF	atrial fibrillation	lncRNA	long non-coding RNAs
AGEs	advanced glycation end products	MAPK	mitogen-activated protein kinase
AI	artificial intelligence	MCP-1	monocyte chemoattractant protein-1
ALR	adiponectin/leptin ratio	MFG-E8	milk fat globule-EGF factor 8
AMPK	Amp-activated protein kinase	miRNA	microRNA
BAT	brown adipose tissue	MRI	magnetic resonance imaging
BCAAs	branched-chain amino acids	NF-κB	nuclear factor kappa-B
BMI	body mass index	NLRP3	nod-like receptor family, pyrin domain containing 3
CT	chest computed tomography	NO	nitric oxide
CVDs	cardiovascular diseases	PET-CT	positron emission tomography-computed tomography
DCM	diabetic cardiomyopathy	PPAR	peroxisome proliferator-activated receptor
EAT	epicardial adipose tissue	PVAT	perivascular adipose tissue
eNOS	endothelial nitric oxide synthase	RAAS	renin–angiotensin–aldosterone system
EVs	extracellular vesicles	ROS	reactive oxygen species
FABP4	fatty acid-binding protein 4	RYGB	Roux-En-Y gastric bypass
FFAs	free fatty acids	SCFAs	short-chain fatty acids
FGF21	fibroblast growth factor 21	SLGT2	sodium-glucose cotransporter 2
GLP-1RAs	glucagon-like peptide-1 receptor agonists	SOCS3	suppressor of cytokine signaling 3
HF	heart failure	TGF-β	transforming growth factor-beta
HFpEF	HF with preserved ejection fraction	TMAO	trimethylamine N-Oxide
HFrEF	heart failure with reduced ejection fraction	TNF-α	tumor necrosis factor-α
IL-1β	Interleukin-1β	VAT	visceral adipose tissue
IL-6	Interleukin-6	VSMC	vascular smooth muscle cell
JAK2	Janus Kinase 2	WAT	white adipose tissue
